# Duodenal Adenocarcinoma Metastatic to the Breast: A Case Report

**DOI:** 10.1097/MD.0000000000003088

**Published:** 2016-03-18

**Authors:** Haibo Yu, Hongliang Song, Yi Jiang

**Affiliations:** From the Department of Surgery (HY, HS); and Department of Pathology (YJ), The Dingli Clinical Institute of Wenzhou Medical University (Wenzhou Central Hospital), Da Jian Lane 32, Wenzhou, Zhejiang, Republic of China.

## Abstract

Duodenal adenocarcinoma, a very rare malignant gastrointestinal tumor, mainly metastasizes via the lymphatic system. Metastases from duodenal adenocarcinomas to the breast are very uncommon.

A 31-year-old woman presented at our department with a left breast tumor. She had a past medical history of duodenal adenocarcinoma. Physical examination on admission confirmed a 2.5-cm-diameter tumor in the outer lower quadrant of the left breast. Computed tomography (CT) examination showed a soft lesion with tissue-like density and enlarged axillary lymph nodes. Local excision was performed to remove the breast lesion. The findings of cytologic, histologic, and immunohistochemistry examination indicated a breast metastasis from the previous duodenal adenocarcinoma. The patient was treated with palliative chemotherapy.

Metastases from duodenal adenocarcinoma to the breast are rare. The diagnosis depends on medical history, imaging, and pathologic examination including immunohistochemistry. An accurate diagnosis is important to avoid unnecessary surgery.

## INTRODUCTION

Duodenal adenocarcinoma is rare: although most small bowel adenocarcinomas (56%) arise in the duodenum, small bowel cancers account for only 2% of all gastrointestinal cancers in the United States.^1,2^ It mainly metastasizes via the lymphatic system; lymph node metastases are associated with a poor prognosis.^3^ Here, we report a case of a young woman who we diagnosed as having a breast metastasis from a duodenal adenocarcinoma.

## CASE PRESENTATION

**FIGURE 1:**
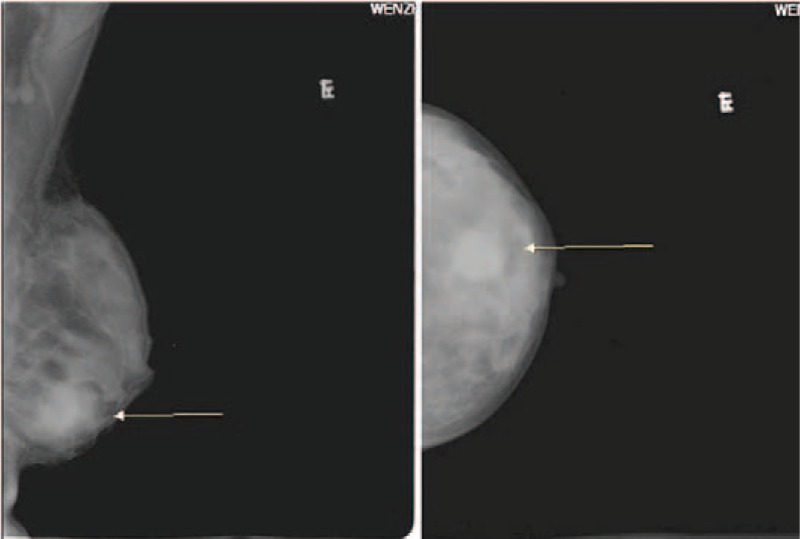
Mammography showed a hyperdense mass with a circumscribed border.

**FIGURE 2:**
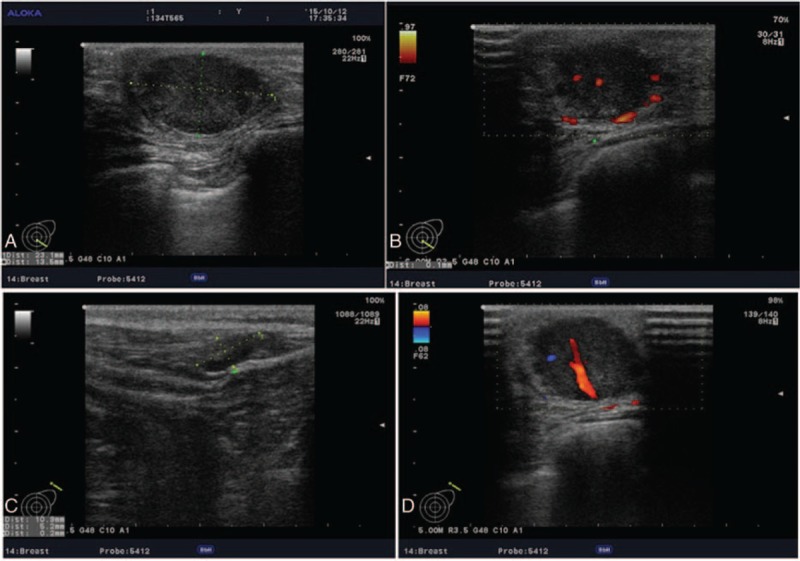
Ultrasound showed a 23-mm × 14-mm nodular low echo in the outer lower quadrant of the left breast with a non-uniform texture and a well-defined boundary. Color Doppler flow images displayed blood flow. Multiple axillary lymph nodes were identified; the largest measuring 11 mm × 5 mm.

**FIGURE 3:**
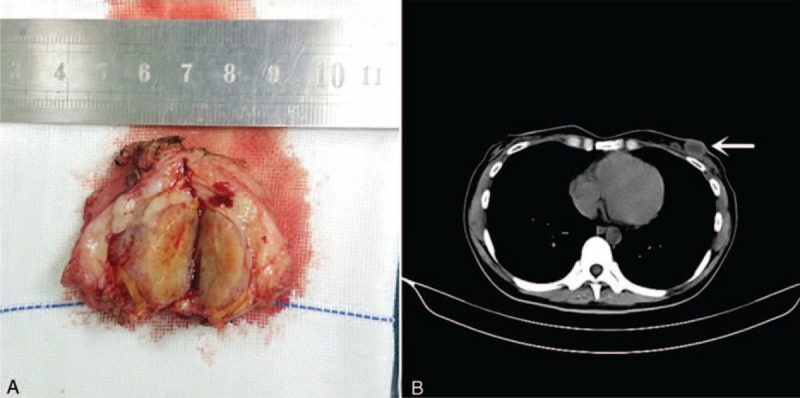
A. Gross examination showed the breast lesion with the diameter of 2.5 cm in the outer lower quadrant of the left breast. B. Computed tomography examination showed a soft 22-mm × 17-mm lesion of tissue-like density with a central patchy low-density area in the lower quadrant of the left breast with a well-defined boundary.

**FIGURE 4:**
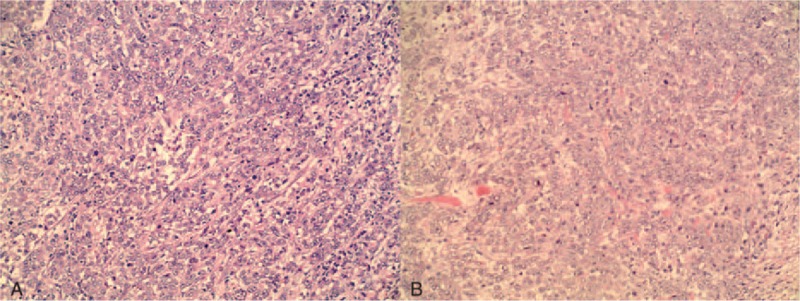
A. Metastatic duodenal adenocarcinoma to the left breast (H&E, 200×). B. Duodenal adenocarcinoma (H&E, 200×).

**FIGURE 5:**
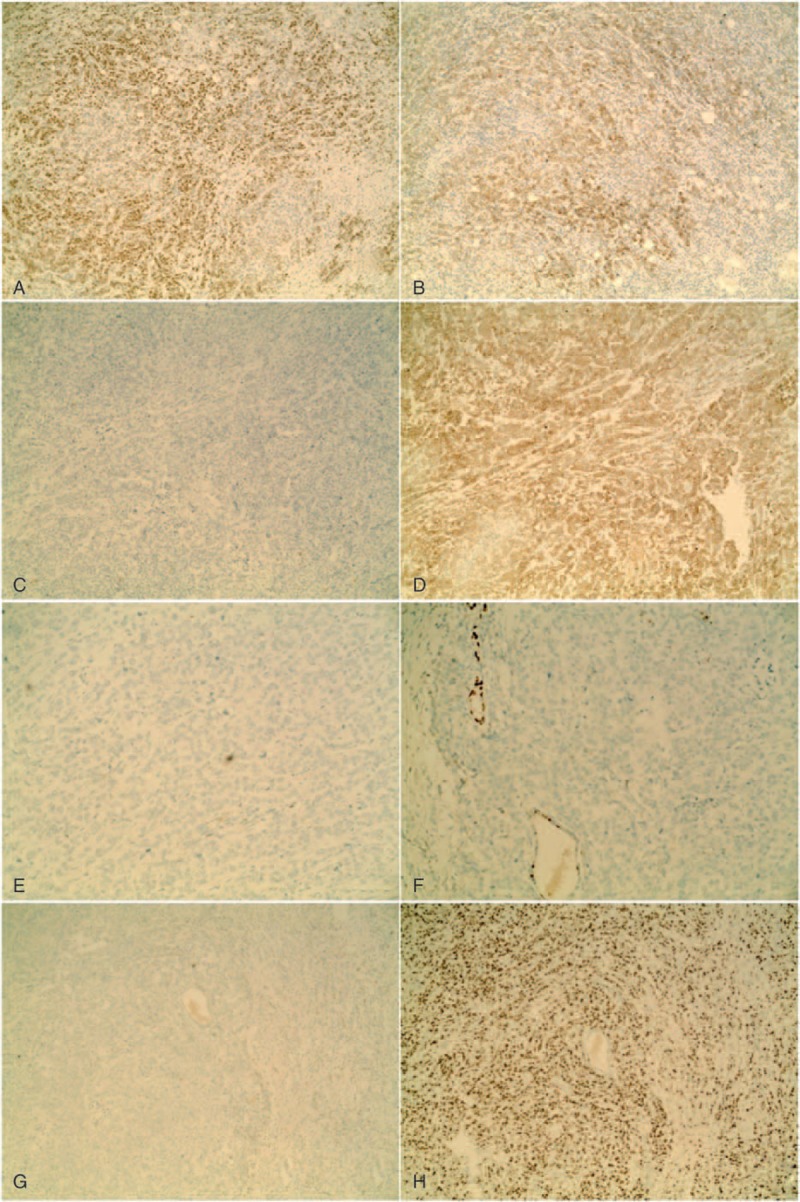
Immunohistochemical analysis revealed the mammary tumor cells were positive for CDX2 (A), villin (B), cytokeratin 20 (C), and cytokeratin 7 (D), but negative for estrogen receptors (E) and progesterone receptors (F) and human epidermal growth factor receptor-2 (G); 70% of the cells were Ki-67 positive (H).

A 31-year-old woman was admitted to our hospital because of a left breast tumor. Her family history was noncontributory. She had undergone pancreaticoduodenectomy for a duodenal adenocarcinoma 18 months previously and had received 6 courses of postoperative chemotherapy. A chest CT examination 4 months prior to the current presentation had shown no abnormalities. Physical examination revealed a 2.5-cm-diameter tumor in the outer lower quadrant of the left breast. Mammography showed a hyperdense mass with a circumscribed border (Figure 1) and ultrasound showed a 23-mm × 14-mm nodular low echo in the outer lower quadrant of the left breast with a non-uniform texture and a well-defined boundary. Color Doppler flow images displayed blood flow. Multiple axillary lymph nodes were identified; the largest measuring 11 mm × 5 mm (Figure 2). CT examination showed a soft 22-mm × 17-mm lesion of tissue-like density with a central patchy low-density area in the lower quadrant of the left breast with a well-defined boundary. Lymph nodes were identified in the left axilla (Figure 3B). Local excision was performed to remove the breast lesion (Figure 3A), the provisional diagnosis being invasive carcinoma. Pathologic examination of the resected specimen showed poorly differentiated adenocarcinoma (Figure 4A). Immunohistochemical analysis revealed that mammary tumor cells were positive for CDX2 (Figure 5A), villin (Figure 5B), cytokeratin 20 (Figure 5C), and cytokeratin 7 (Figure 5D), but negative for estrogen receptors (Figure 5E) and progesterone receptors (Figure 5F) and human epidermal growth factor receptor-2 (Figure 5G); 70% of the cells were Ki-67 positive (Figure 5H). Comparison of the pathological findings with those of the previous duodenal adenocarcinoma (Figure 4B) resulted in a final diagnosis of a metastasis to the breast from duodenal adenocarcinoma. The patient was then treated with palliative chemotherapy. At the time of submission of our manuscript, the patient was tolerating the new chemotherapy regimen well and had been followed for 2 months after a definitive diagnosis had been made.

## DISCUSSION

Breast cancer is the commonest type of malignant tumor in women; however, metastases to the breast from extramammary malignancies are very rare, accounting for only 0.43% of all malignant breast tumors.^4^ The most common sources of such metastases are malignant melanoma, pulmonary, ovarian, renal, cervical, prostate, stomach, thyroid, and colorectal carcinomas, and small bowel carcinoid tumors.^5–9^ To the best of our knowledge, this is the first reported case of duodenal adenocarcinoma metastasizing to the breast. Our patient presented after she had detected a breast mass. A chest CT examination 4 months previously had not shown a breast tumor. She had a history of resection of a duodenal carcinoma. We, therefore, strongly suspected that the breast tumor might be metastatic carcinoma. However, ultrasound and mammography did not suggest a malignant tumor. The patient underwent resection of the breast tumor and comparison of the pathological sections of the excised specimen with those of the previous duodenal adenocarcinoma resulted in a diagnosis of a metastasis from the duodenal carcinoma to the breast. The correct diagnosis was, therefore, crucial in avoiding unnecessary surgical interventions.

A diagnosis of metastasis to the breast must be confirmed by pathologic examination. When a patient's history reveals a previous primary malignancy, the pathological characteristics, including immunohistochemical findings, of a breast lesion should be compared with those of the previous primary tumor. A specific diagnosis cannot be made by imaging procedures, such as mammography, ultrasound or CT. On mammography, breast metastases reportedly present as round lesions with smooth margins.^10^ The imaging findings in our case suggested a benign tumor; however, pathological examination resulted in a diagnosis of metastatic poorly differentiated adenocarcinoma. Thus, the final diagnosis depends on surgical resection or biopsy. Breast cancer can metastatic to the duodenum,^11^ there might be some relationship between the 2 organ, but the mechanism is not yet understood and needs further study.

## CONCLUSIONS

Our patient illustrates that metastasis to the breast from duodenal adenocarcinoma does occur. Despite its rarity, it should be considered in the differential diagnosis of an apparent primary mammary carcinoma because the treatment and prognosis differ significantly. Those working in the field of breast diagnosis and therapy should be aware of the possibility of metastases to the breast.
